# High early strength concrete incorporating waste derived nanomaterials for sustainable construction

**DOI:** 10.1038/s41598-024-81178-4

**Published:** 2024-12-23

**Authors:** Nehal Hamed, M. I. Serag, M. M. El-Attar, M. S. El-Feky

**Affiliations:** 1 Department of Structural Engineering, Faculty of Engineering, Cairo University, Giza, Egypt; 2 Department of Civil Engineering, The Higher Institute of Engineering and Technology Fifth Settlement, New Cairo, Egypt; 3 Department of Civil Engineering, National Research Centre, Dokki, Egypt

**Keywords:** Nanomaterials, High early strength concrete, Waste-driven, Nano-clay, Nano-silica, Nano-cellulose, Full factorial design, Mechanical properties, Microstructure, Sustainable construction, Circular economy, Engineering, Materials science, Nanoscience and technology

## Abstract

This paper contributes to the expanding knowledge base on nanomaterial-enhanced cementitious composites, offering valuable insights for developing high-performance, sustainable concrete solutions. The study assessed the effects of three different types of nanomaterials—nano clay (NC), nano silica (NS), and nano cellulose (NCel)—on the compressive strength of high-early-strength concrete (HESC) through both experimental studies and a 2^3^ factorial design. Incorporating nanomaterials into the HESC matrix led to a decrease in workability, with NCel demonstrating the least impact on this property across all studied replacement percentages. All HESC mixes containing nanomaterials exhibited higher compressive strength than the control mix (M mix) across all ages. The optimal percentages for compressive strength enhancement were 4.5% NC (33.43% increase at 3 days, 22.29% at 7 days, and 12.15% at 28 days), 4.5% NS (20.12%, 11.14%, and 4.89% respectively), and 0.0375% NCel (34.91%, 25.76%, and 13.46% respectively). The highest compressive strength was observed in the hybrid mix containing 4.5% NC and 0.0375% NCel, yielding strength enhancements of 35.7%, 26%, and 12.75% compared to the M mix. Statistical analysis indicated that nano cellulose had the most significant contribution to enhancing compressive strength, followed by nano clay. The mathematical models derived from the statistical analyses provide a reliable means of predicting the compressive strength of HESC at 3, 7, and 28 days based on nanomaterial content. Contour plots illustrated the optimization of compressive strength across different nanomaterial contents at each age. In summary, the findings underscore the potential of waste-derived nanomaterials to enhance the performance of HESC, paving the way for innovative waste utilization strategies in construction. The study emphasizes the importance of reducing curing times, improving structural durability, and minimizing the environmental impact associated with concrete production.

## Introduction

The utilization of high-strength concrete (HSC) and ultra-high-strength concrete (UHSC) in lieu of conventional concrete has become increasingly prevalent in the construction industry. This trend is primarily driven by the advantageous high strength-to-weight ratio of these advanced cementitious composites, which enables the construction of high-rise structures with smaller structural elements^1,2^. However, a significant challenge associated with these high-strength concretes is the extended time required for formwork removal and strength development. To address this issue, the incorporation of early-strength concrete (ESC) has emerged as a viable solution, allowing for reduced construction timelines while maintaining the desirable mechanical properties of small-sized structural elements^1,2^.

The incorporation of waste and renewable materials, such as silica fume, metakaolin, and fly ash, in concrete has been extensively researched, demonstrating their potential to produce eco-friendly construction materials with enhanced early-stage strength and reduced costs^3–12^. In parallel, the application of nanotechnology has gained widespread attention in the construction field^13–19^. Nanotechnology involves the restructuring of materials at the nanoscale (< 150 nm) to develop novel properties and functionalities^20^. The use of nanomaterials as partial cement replacements in concrete can significantly improve its performance, as the primary hydration product, calcium-silicate-hydrate (C–S–H) gel, is a nanostructured material^21^. Additionally, the presence of a multitude of nano-sized pores in cement matrices renders concrete a material that is influenced by its nano-scale characteristics^22^.

Nanomaterials can be classified into two main categories: (1) pozzolanic materials, such as nano-silica and nano-clay, which undergo pozzolanic reactions with calcium hydroxide (CH) from cement hydration, producing additional C–S–H gel and enhancing matrix strengths^22–28^; and (2) fiber-like materials, such as carbon nano-fibers, carbon nanotubes, and nano-cellulose, which exhibit a needle-like action that can bridge and resist the propagation of cracks, thereby improving the tensile properties of the concrete matrix^27–32^.

Nano-silica (NS) is a widely used sustainable material with a high surface area that contributes to the production of high-early-strength concrete through three primary mechanisms: (1) the filling effect of NS particles due to their nano-size, which densifies the concrete matrix and improves compaction^23–25,33–37^; (2) the pozzolanic reactivity of NS particles, which reacts with excess CH to form additional C–S–H gel, enhancing the interfacial transition zone (ITZ) between aggregates and cement blends, thereby promoting strength^23–25,33–39^; and (3) the nucleation effect of NS particles, which acts as a catalyst for the formation of reinforced C–S–H^3,24,25,33,35^. Nano-clay (NC) is another sustainable material used in concrete to enhance early strength, primarily due to its four main performances: (1) pozzolanic reactivity^22,26,40–43^; (2) filling effect due to its nano-sized particles^22,26,40,41,43^; (3) nucleation effect, acting as a nucleus for cement hydration phases^22,26,40–42^; and (4) needle-like action system, which can reinforce the cement component and retard the progress of microcracks^22,26,41^. Nano-cellulose (NCel) is a renewable, hydrogel-shaped material produced from cellulose fibers through a mechanical disintegration process^44,45^. Research has shown that the addition of nano-cellulose to concrete can enhance its early properties by acting as a nucleus for cement hydration products, filling pores to create a compact microstructure, and controlling the progression of microcracks due to the shape of its particles^31,32,46–48^.

Several experimental studies had conducted to study the effect of one of the three types of nanomaterials (NS, NC and NCel) individually or each of the two types together on high early strength concrete, There was a contradiction in previous researches on determining the optimum percentages of each type of nanomaterials to promoting the compressive strength of high early strength concrete. In this paper, to study the main effects of cement substitution percentages by each type of the three nanomaterials (NS% up to 4.5%, NC up to 4.5% and NCel% up to 0.0375%) as separately or as a hybrid on the compressive strength of HESC at different ages of 3, 7 and 28 days and to study the interaction between these three nanomaterials, the full factorial designed experiment was chosen to use in this study. A full factorial design is a simple method to design an experiment with independent variables at different levels to estimate the main effects of each variable on the studied response, the interactions between these variables depending on the response and finally obtain the level for each variable that optimize the response^49–52^. The full factorial design used in this paper is 2^k^ factorial design, which is the simplest type of factorial design that has two levels for each factor with a high and low value, where k is the number of factors^51,52^. In this study, the effect of the cement substitution by three types of nanomaterials of nano clay (NC), nano silica (NS) and nano cellulose (NCel) either as separately or as hybrid on the compressive strength of high early strength concrete at different ages of 3, 7 and 28 days, the 2^3^ factorial design was used for the experimental design, where the three types of nanomaterials were used as three factors with two levels for each type of nanomaterial and the responses were the compressive strength at different ages 3, 7 and 28 days.

Understanding the effects of these nanomaterials on HESC is crucial for the development of sustainable and high-performance construction materials. For instance, Nano silica as a well-studied, readily available ultra-fine mineral admixture (UFMA) featuring nanoscale particles for high-performance cement composites. Often utilized in small amounts within ternary or quaternary blend systems, nano silica is assessed for its ability to enhance early strength^23,53–62^. When used in optimal quantities, it exhibits an accelerating effect in cement paste, leading to early strength gains^55,60,61^. The addition of nano silica has been shown to improve the microstructure of concrete by refining the pore structure in hydrated phases, thereby enhancing the overall properties of the concrete^23,57,58^. nano silica also demonstrates a synergistic effect when combined with various cementitious and pozzolanic materials, including other ultra-fine mineral admixtures^23,58,59^. By examining the individual and combined influences of nano silica, nano clay, and nano cellulose on the compressive strength of HESC at various ages, researchers and practitioners can optimize the use of these nanomaterials to achieve desired early strength characteristics. This knowledge can drive advancements in sustainable construction practices, shorten construction timelines, and facilitate the creation of innovative concrete solutions that meet the demands of modern infrastructure projects.

What sets these nanomaterials apart is their local production and derivation from industrial waste streams. Nano silica is extracted from rice husk, a byproduct of the agricultural industry, while nano cellulose is derived from sawdust waste. Additionally, nano clay is sourced from local kaolinite deposits. This approach ensures the sustainability and eco-friendliness of the construction materials by minimizing reliance on virgin raw materials and promoting the utilization of waste products. Consequently, the environmental impact associated with primary resource extraction and processing is reduced, contributing to the circularity of the construction industry.

In summary, studying the effects of nanomaterials on high early strength concrete holds great promise for the development of sustainable, high-performance construction materials. The use of locally produced, waste-derived nanomaterials not only enhances the early strength characteristics of HESC but also drives advancements in sustainable construction practices, reduces construction timelines, and facilitates the creation of innovative concrete solutions that meet the evolving demands of modern infrastructure projects.

## Experimental procedure

### Properties of used materials

**Fig. 1:**
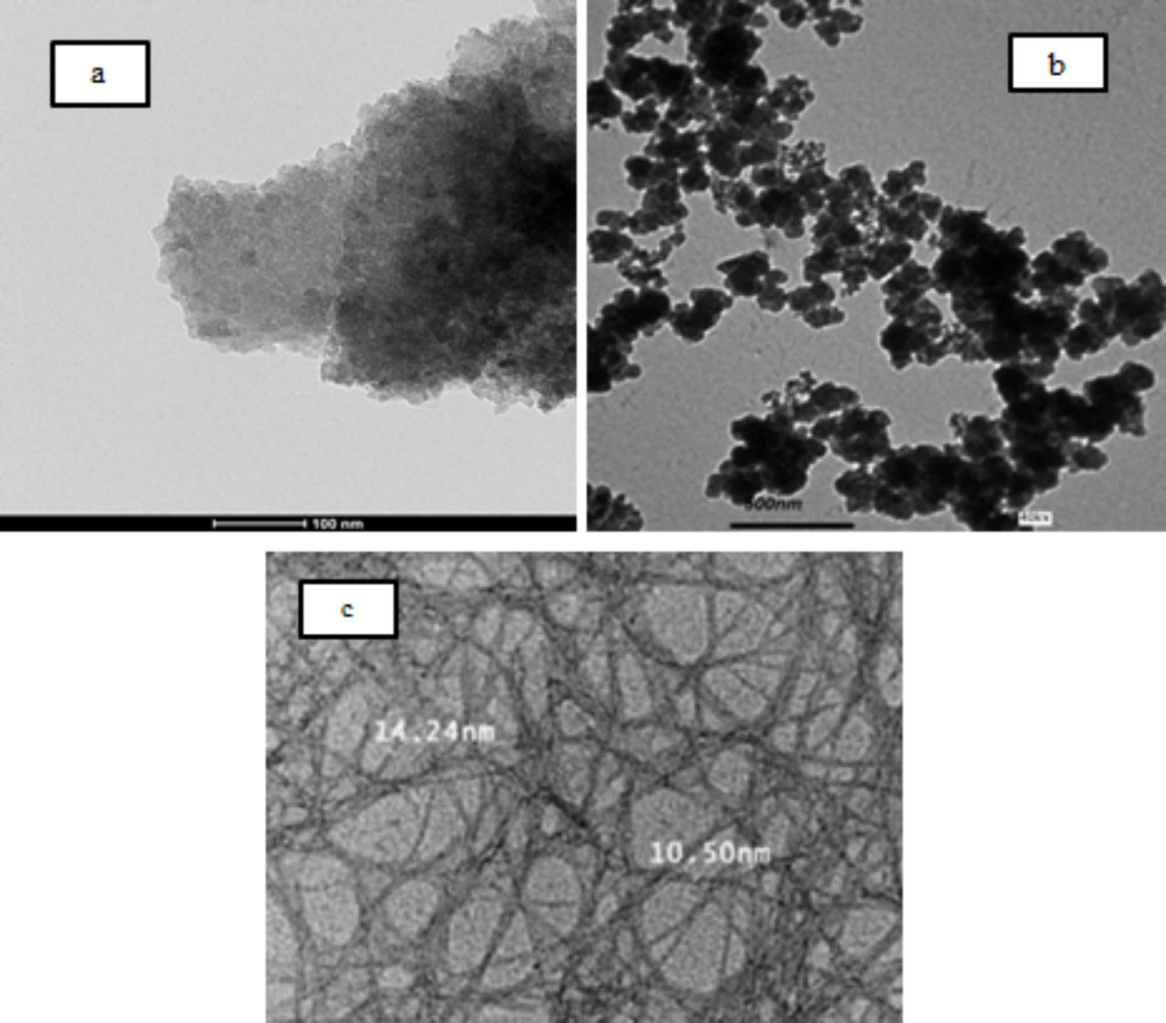
The TEM micrographs for; (**a**) Nano-clay, (**b**) Nano-silica and (**c**) Nano cellulose.

Commercially available Ordinary Portland cement 52.5 grade conforming to ASTM C150^63^ was used, Sika-Fume (SF) used as an additive in fine-powder form based on silica fume technology, three different types of nanomaterials were used in this research; nano clay, nano silica and nano cellulous. The nano-clay (NC) used is an off-white powder with particles size less than 100 nm. The nano-silica (NS) used is a white powder with average particles size of 20 to 80 nm. The nano cellulose (NCel) used is a dispersed gel in water with concentration of 5% nano cellulose. Figure 1 shows the TEM micrograph of the three different nanomaterials used in this paper and the chemical compositions of cement, SF, NC and NS are presented in Table 1. As for the nano cellulose it primarily consists of cellulose, with the chemical formula (C6H10O5)n, where n represents the number of repeating glucose units. Natural sand is used as fine aggregates with specific gravity of 2.58 g/cm^3^, Crushed clean dolomite is used as coarse aggregate with maximum size of 10 mm and specific gravity of 2.96 g/cm^3^. The mixtures aggregates consist of an incorporation of sand and crushed dolomite with the percentage of 40% and 60% by weight respectively. The superplastizer (S.P) used is viscoCrete-4325; it is a brown liquid solution of modified polycarboxylates with density of 1.08 kg/Lt.

**Table1:** The chemical composition of cement, SF, NC and NS.

Components	SiO_2_	Al_2_O_3_	Fe_2_O_3_	Cao	MgO	SO_3_	Na_2_O	K_2_O	TiO_2_	P_2_O_5_	L.O.I
C	20.13	5.32	3.61	61.63	2.39	2.87	0.37	0.13	–	–	1.96
NC	61.24	20.89	1.06	0.16	0.22	–	0.71	–	0.13	–	13.12
NS	99.17	0.13	0.06	0.14	0.11	–	0.4	–	–	0.01	–
SF	97.0	0.2	0.5	0.2	0.5	0.15	0.2	–	–	–	–

### Experimental design

#### The separate effect of each type of utilized nanomaterials on HESC

Ten mixes were prepared to study the effect of using three different types of nanomaterials (NS, NC and NCel) separately as a partial replacement for cement weight on the workability and the compressive strength of high early strength concrete (HESC). The control mix is prepared of cement (C) sikaFume (SF), coarse aggregate (C.A), fine aggregate (F.A), water (W) and superplastizer (S.P), while the nanomaterials mixes are prepared in the same manner but by three different types of nanomaterials with different percentages of (0.0, 1.5%, 3% and 4.5%) NS, (0.0, 1.5%, 3% and 4.5%) NC and (0.0%, 0.0125%, 0.025%, 0.0375%) NCel. The mix proportions of the ten HESC mixes are shown in Table 2.

**Table 2:** Mix proportions of HESC mixes (kg/m^3^).

Mix	C	SF	F.A	C.A	W	S.P	NC	NS	NCel
M	600	90	635	960	160	9	–	–	–
1.5% NC	591	90	635	960	160	9	9	–	–
3%NC	582	90	635	960	160	9	18	–	–
4.5%NC	573	90	635	960	160	9	27	–	–
1.5%NS	591	90	635	960	160	9	–	9	–
3%NS	582	90	635	960	160	9	–	18	–
4.5%NS	573	90	635	960	160	9	–	27	–
0.0125%NCel	598.5	90	635	960	160	9	–	–	1.5
0.025%NCel	597	90	635	960	160	9	–	–	3
0.0375%NCel	595.5	90	635	960	160	9	–	–	4.5

#### The hybrid effect of the utilized nanomaterials on HESC

The sixteen experiments have been designed by using the 2^3^ factorial designs in Minitab 18 statistical software, the studied variables were the type of nanomaterials with two replacement levels of (0% and 4.5%) NC, (0% and 4.5%) NS and (0% and 0.0375%) NCel, where the factorial design replicated twice was used to fit the full interactions model for the compressive strength (F_C_) as a response to the selected variables (the amount of nanomaterials added). The experiments design is shown in Table 3.

**Table 3:** Experiments design by 2^3^ factorial design.

Run	Nanomaterials % (as a partial replacement of cement weight)		
	NC	NS	NCel
M1	0.0	4.5	0.0000
M2	4.5	4.5	0.0375
M3	0.0	0.0	0.0000
M4	0.0	4.5	0.0375
M5	4.5	0.0	0.0375
M6	4.5	0.0	0.0000
M7	0.0	0.0	0.0375
M8	4.5	0.0	0.0375
M9	4.5	0.0	0.0000
M10	4.5	4.5	0.0000
M11	4.5	4.5	0.0375
M12	0.0	4.5	0.0375
M13	0.0	0.0	0.0000
M14	0.0	0.0	0.0375
M15	0.0	4.5	0.0000
M16	4.5	4.5	0.0000

Analysis of Variance (ANOVA) is used to analyze the main effects of the three variables of nanomaterials percentages (NC, NS and NCel) in the compressive strength of high early strength concrete. The equation obtained from the statistical analysis of the model to represent the predicted value for studied response is stated as shown in Eq. (1)

1 $$\begin{aligned} {\text{FC }} & = { }a_{0} + a_{1} X_{1} + a_{2} X_{2} + a_{3} X_{3} + a_{12} X_{1} X_{2} \\ & \quad + a_{23} X_{2} X_{3} + a_{13} X_{1} X_{3} + a_{123} + X_{1} X_{2} X_{3} \\ \end{aligned}$$

where F_C_ is the predicted compressive strength, ($${X}_{1}$$, $${X}_{2}$$ and $${X}_{3}$$) are the nanomaterials percentages according to its type, ($${X}_{1}{X}_{2}$$, $${X}_{2}{X}_{3}$$ and $${X}_{1}{X}_{3}$$) are the interaction terms of each two nanomaterials together, ($${X}_{1}$$ $${X}_{2}$$ $${X}_{3})$$ is the interaction term of all used nanomaterials together, $${a}_{0}$$ is the constant coefficient about numerical value, ($$a_{1} , a_{2} \;and\; a_{3}$$) are the linear coefficients of each type of the used nanomaterials, $$\left( {a_{12} ,a_{23} \;and\; a_{13} } \right)$$ are the interaction coefficients between each two types of the used nanomaterials and $$({a}_{123})$$ is the interaction coefficient between all used types of nanomaterials together. From the Eq. (1); when found the value of $${a}_{i}$$ is larger, it is means that the contribution of the variable $${X}_{i}$$ to the response F_C_ is greater.

### Preparation of the specimens, curing and tests

The mix proportions for 1 m^3^ of high early strength concrete (HESC) was consists of 600 kg cement, 90 kg sikaFume, 960 kg coarse aggregate, 635 kg fine aggregate, 9 lt superplastizer, 160 lt water and different percentages of nanomaterials types as partial replacement by cement weight according to Tables 2 and 3. The procedure of mixing was started with mixing the dry materials in the mixer for 1.5 min, then adding 75% of mixing water for all concrete mixes, and mixing for another 1 min., and finally, adding the superplastizer with the rest of the mixing water and mixing for another 1 min. and once the concrete mixing process was finished, the workability was measured by slump test on the fresh concrete according to ASTM C143^64^. Cubes of 100 × 100 × 100 mm^3^ were prepared and cast for implementing compressive strength test after 3,7, and 28 days of water curing according with BS EN 12390-3^65^.

## Results and discussion

### The separate effect of each type of utilized nanomaterials on HESC

#### Workability

**Fig. 2:**
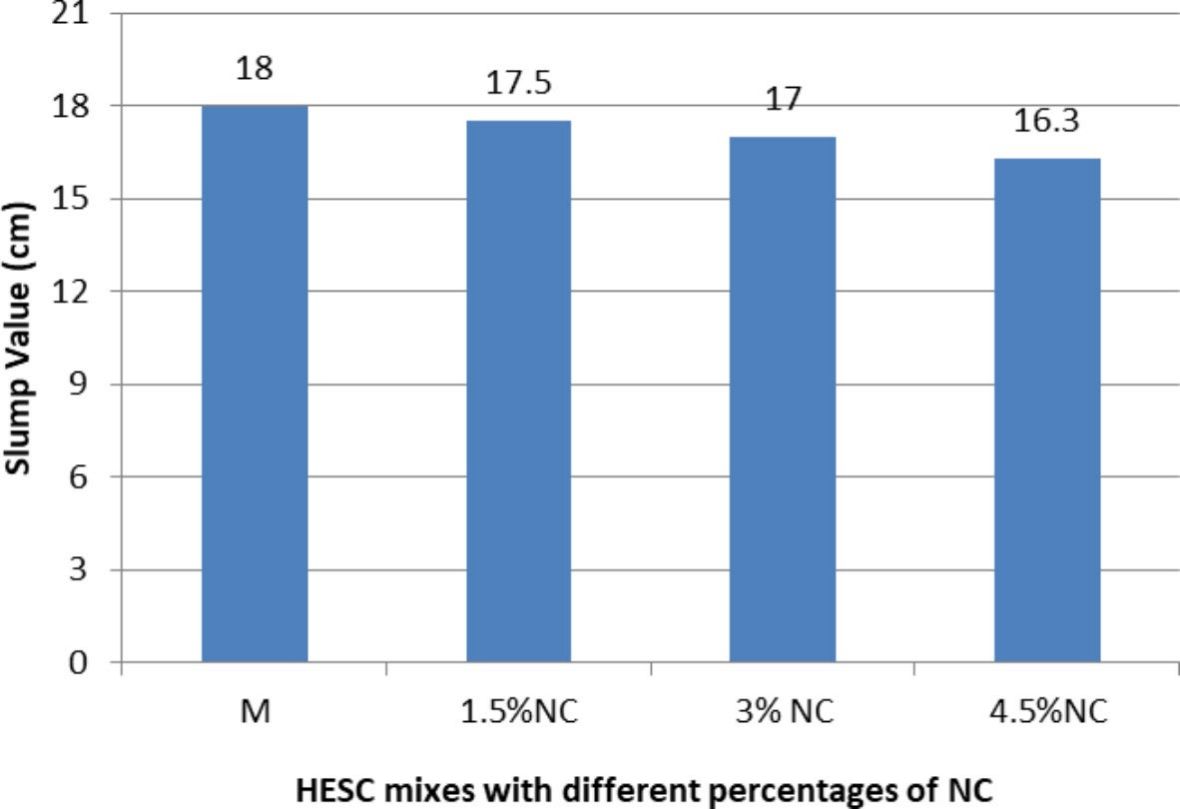
The slump value of HESC mixes with different percentage of NC.

**Fig. 3:**
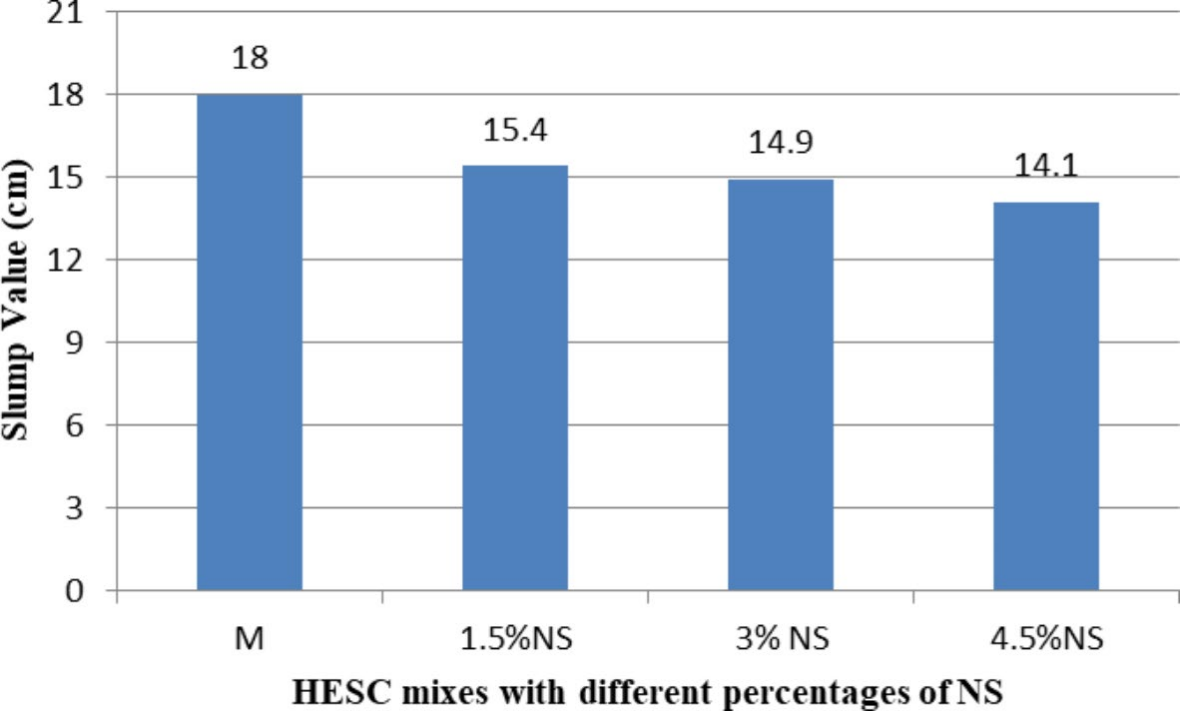
The slump value of HESC mixes with different percentage of NS.

**Fig. 4:**
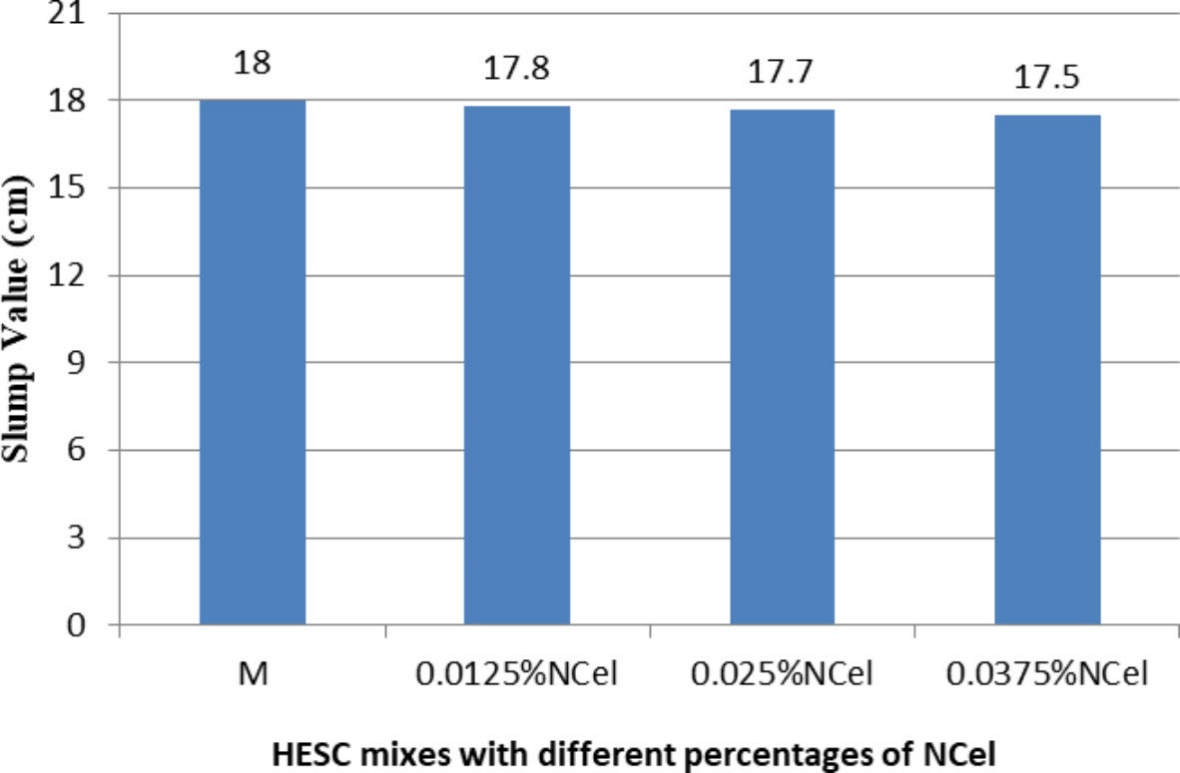
The slump values of HESC mixes with different percentage of NCel.

The workability of High-Early-Strength Concrete (HESC) mixes containing different types of nanomaterials (NC, NS, and NCel) was measured by conducting the slump test on fresh concrete, as shown in Figs. 2, 3 and 4. Generally, increasing the cement replacement percentages with all studied nanomaterials types in HESC mixes led to a decline in their workability.

Figure 2 presents the slump value results of HESC mixes with varying percentages of NC. The slump values were 17.5 cm, 17 cm, and 16.3 cm for cement replacement percentages of 1.5%, 3%, and 4.5% by NC, respectively, compared to 18 cm for the control mix (M mix) without nanomaterials. This represents a decrease of 2.78%, 5.56%, and 9.44%, respectively, compared to the M mix. Increasing the cement replacement percentages by NC led to a slight reduction in the workability compared to the M mix, which can be attributed to the presence of agglomerates due to poor dispersion that trapped a portion of the mixing water inside these agglomerates. The agglomerates increased with increasing the replacement percentages^22^, in addition to the NC’s high water absorption properties, resulting in decreased workability with increasing NC replacement percentages^22,31^.

Figure 3 shows the slump value results of HESC mixes with varying percentages of NS. The slump values were 15.4 cm, 14.9 cm, and 14.1 cm for cement replacement percentages of 1.5%, 3%, and 4.5% by NS, respectively, compared to 18 cm for the M mix. This represents a significant decrease in slump values by 14.44%, 17.22%, and 21.67% respectively compared to the M mix. The workability decreased significantly with increasing the NS replacement percentages, which can be attributed to the poor dispersion of NS particles in large percentages due to their high specific surface area, leading to cohesive forces between the particles and the formation of large NS agglomerates. As a result, the NS agglomerates possess a high water adsorption and significant water retention capacity because of their high specific surface area and high nano-scale porosity^66–68^.

Figure 4 presents the slump value results of HESC mixes with varying percentages of NCel. The slump values were 17.8 cm, 17.7 cm, and 17.5 cm for cement replacement percentages of 1.5%, 3%, and 4.5% by NCel, respectively, compared to 18 cm for the M mix. This represents a slight decrease in slump values by 1.11%, 1.67%, and 2.78%, respectively, compared to the M mix. The workability decreased by a very small amount with increasing the NCel replacement percentages, which can be attributed to the rheology modification effect of nano cellulose. The high aspect ratio coupled with its flexibility promotes the formation of a percolating network of nano filaments, thus increasing the viscosity buildup^69^. Additionally, the use of NCel as a viscous gel has higher dispersion ability in the matrix and reduces the formation of agglomerates^69,70^.

#### Compressive strength

**Fig. 5:**
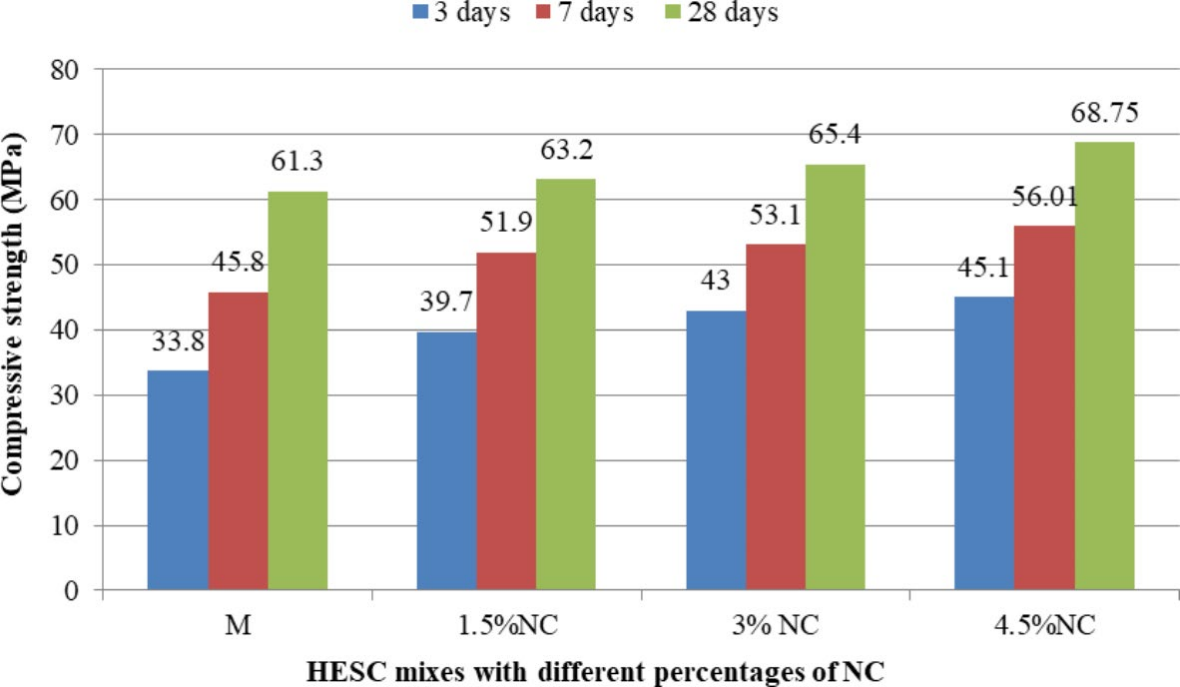
The compressive strength of HESC mixes with different percentage of NC.

**Fig. 6:**
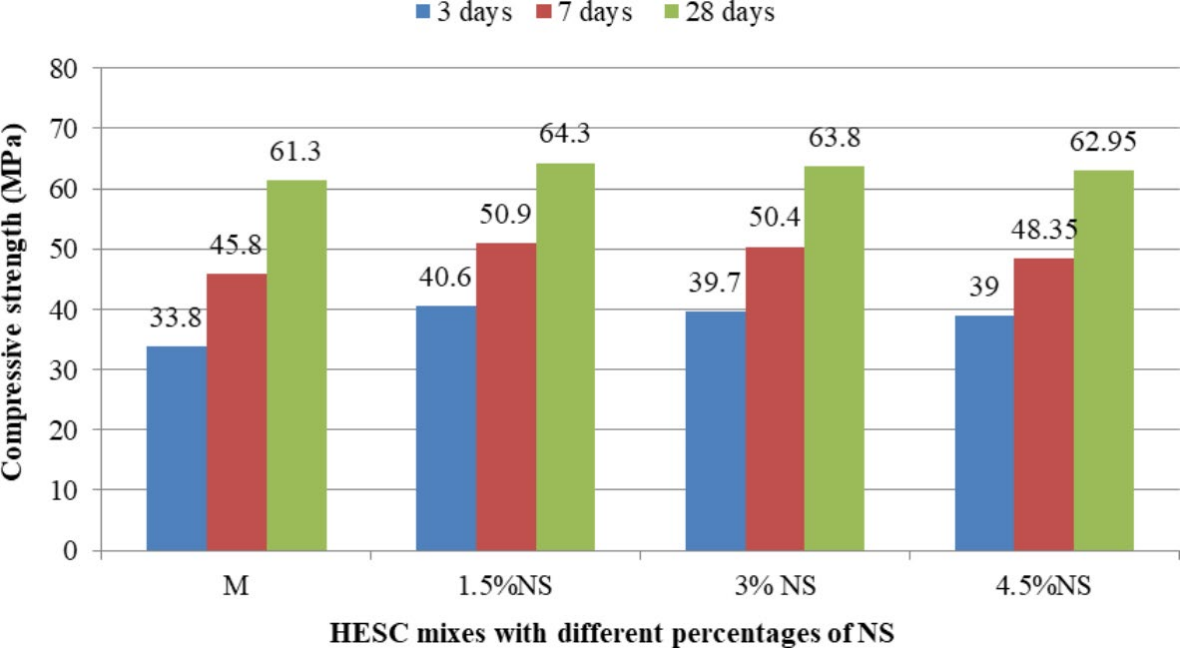
The compressive strength of HESC mixes with different percentage of NS.

**Fig. 7:**
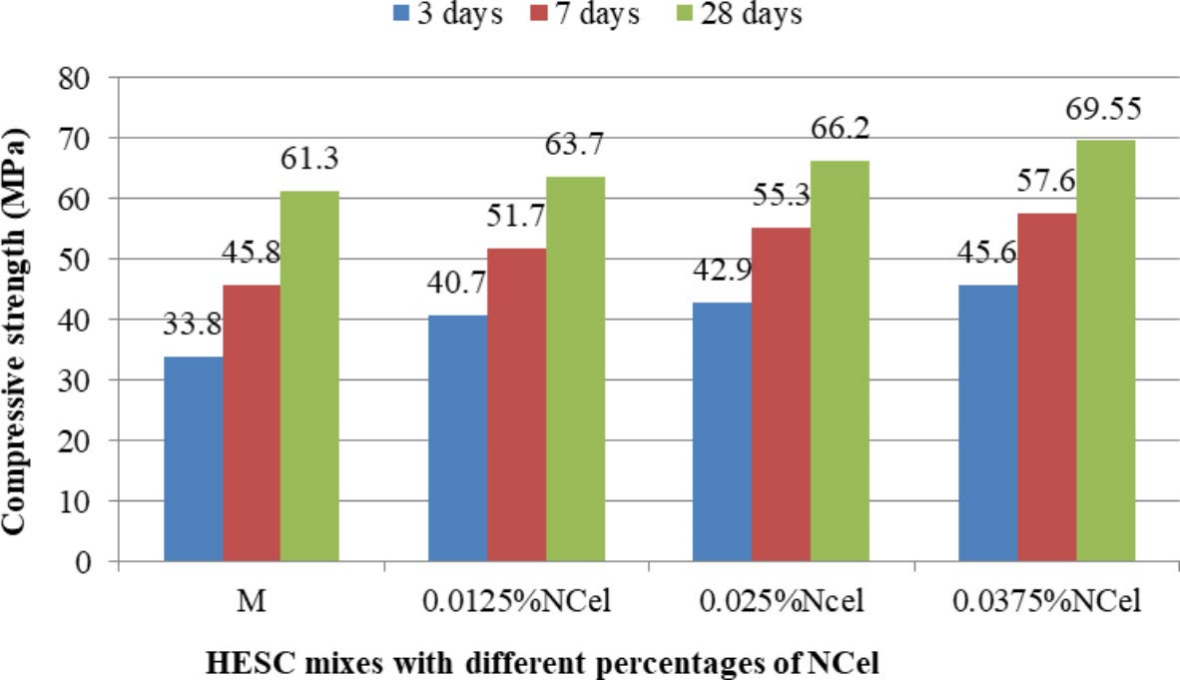
The compressive strength of HESC mixes with different percentage of NCel.

The compressive strength results of HESC mixes containing different types of nanomaterials separately (NC, NS and NCel) at different ages of 3, 7 and 28 days were showed in Figs. 5, 6 and 7. Generally, the use of nanomaterials as a partial replacement for cement led to enhancing the compressive strength of HESC at 3, 7 and 28 days in all studied types of nanomaterials (NC, NS and NCel) and in all studied percentages of each type.

Figure 5 presents the compressive strength results of HESC mixes with different percentages of NC at different ages of 3, 7 and 28 days. The partial cement replacement with NC enhanced the compressive strength at the different ages compared to the M mix (without NC). The compressive strength improvements were increased with increasing the cement replacing with NC. The compressive strength with cement replacement of 4.5%NC reached 45.1 MPa, 56.01 MPa and 68.75 MPa at 3, 7 and 28 days with strength gains of 33.43%, 22.29% and 12.15% respectively compared to M mix without nanomaterials . The enhancement in the compressive strength at early ages of 3 and 7 days can be attributed to the its particles in nano-sized with high surface area that accelerate the cement hydration at early ages, its nucleation effect that acting as a nucleus to the cement hydration phases^22,26,40–42^, pozzolanic reactivity resulting in dense structured matrix^22,26,40–43^, and Needle action system due to its particles shape of a flaky, elongated, thin, and platy which leads to reinforce the cement component and retards the progress of micro cracks^22,26,41^, while at 28 days the enhancements can be attributed to the filling effect due to its particles in nano-sized^22,26,40,41,43^, and Needle action system due to its particles shape of a flaky, elongated, thin, and platy which leads to reinforce the cement component and retards the progress of micro cracks^22,26,41^.

Figure 6 presents the compressive strength results of HESC mixes with different percentages of NS at different ages of 3, 7 and 28 days. The partial cement replacement with NS enhanced the compressive strength at the different ages compared to the M mix (without NS). At the early ages of 3 and 7 days, the presence of NS particles increased the reaction rate due to their high surface area, accelerating cement hydration, and their nucleation effect and pozzolanic reactivity, in agreement with previous studies^35,71^. At 28 days, the enhancement in compressive strength can be attributed to the filling effect of NS, leading to a denser structural matrix^35^. The optimum percentage of NS to enhance compressive strength was 1.5%, with gains of 45.1 MPa, 56.01 MPa and 68.75 MPa at 3, 7 and 28 days, representing strength gains of 20.12%, 11.14% and 4.89% respectively, compared to the M mix without nanomaterials .

Figure 7 shows the compressive strength results of HESC mixes with different percentages of NCel at different ages of 3, 7 and 28 days. The partial cement replacement with NCel enhanced the compressive strength at the different ages compared to the M mix (without NCel). The enhancement of compressive strength of HESC at the early ages of 3 and 7 days with NCel can be attributed to its effects in accelerating cement hydration due to its high surface area that works as a nucleation site for C–S–H, in agreement with previous studies^45,46,72^, and its filling effect, leading to a compact microstructure. At 28 days, the enhancements in compressive strength can be attributed to the bridging effect of NCel particles due to their shapes, which can bridge the hydration products together, promoting the development of mechanical properties^47^. The optimum percentage of NCel to enhance compressive strength was 0.0375%, with gains of 45.6 MPa, 57.6 MPa and 69.55 MPa at 3, 7 and 28 days, representing strength gains of 34.91%, 25.76% and 13.46% respectively, compared to the M mix (without nanomaterials ).

#### Microstructure analysis

**Fig. 8:**
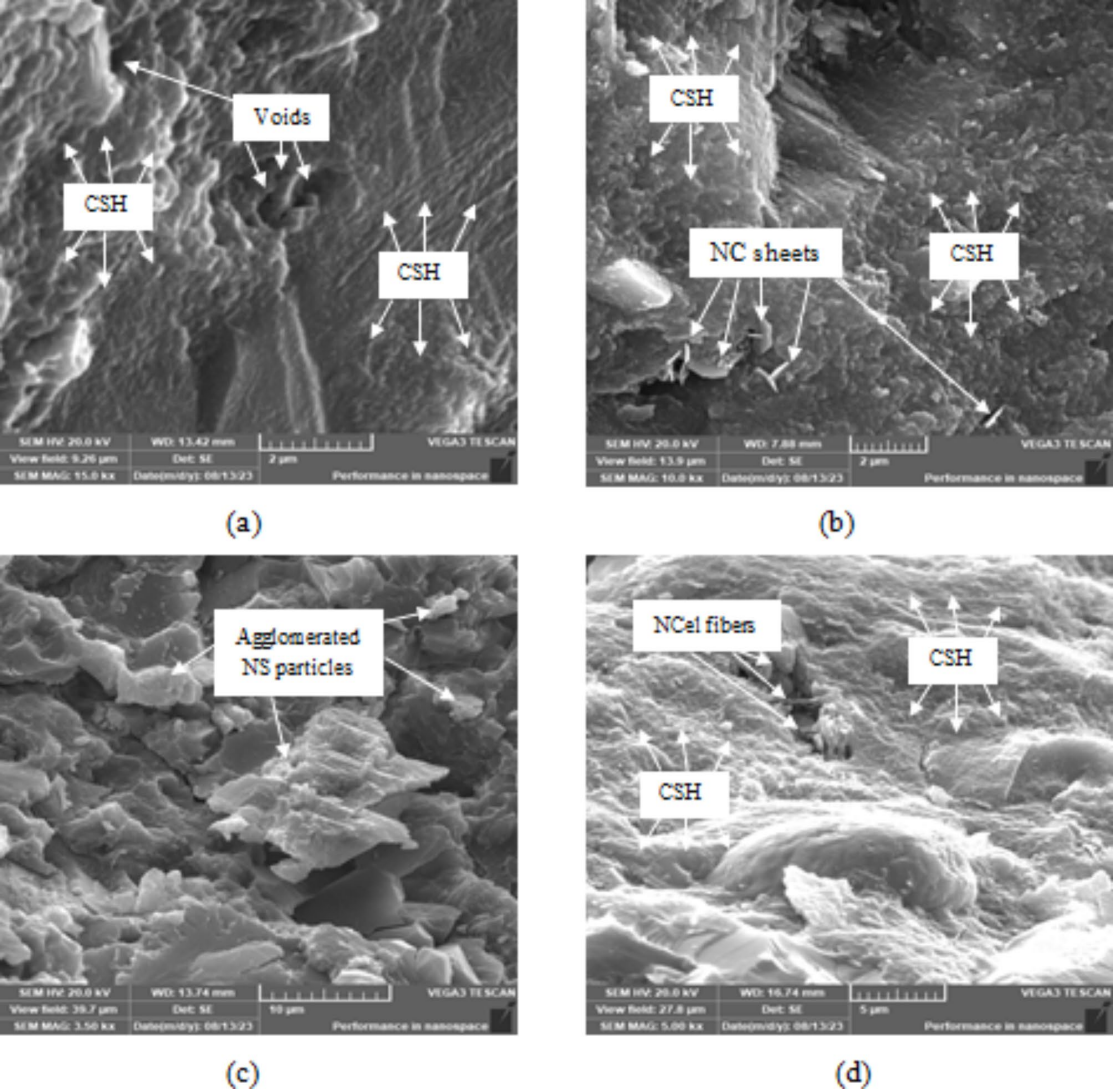
SEM of HESC mixes incorporating nanomaterials; (**a**) M mix (without nanomaterials), (**b**) 4.5% NC mix, (**c**) 1.5% NS mix and (**d**) 0.0375%NCel mix.

Figure 8 shows the scanning electron micrographs (SEM) for the four HESC mixes: the M mix (without nanomaterials), 1.5% NS, 4.5% NC and 0.0375% NCel. The SEM micrographs reveal the morphology and structure of the mixes, providing insights into the previously discussed compressive strength results. The M mix (Fig. 8a) showed a weaker performance in compressive strength compared to mixes incorporating nanomaterials. The presence of nanomaterials led to minimizing the void ratio within the matrix and enhancing the consistency and homogeneity of the matrix, likely due to the filling effect of the nano-sized particles. Figure 8b shows that the C–S–H component was spread within the matrix of the 4.5% NC mix, which can be attributed to the reactivity of NC particles with the residual calcium hydroxide from the cement hydration process, resulting in a higher amount of C–S–H gels. Additionally, the NC sheets acted as bridges, connecting the crack faces and preventing crack propagation. The SEM micrograph of the HESC mix incorporating 1.5% NS (Fig. 8c) revealed groups of unreacted agglomerations of the NS particles, surrounded by C–S–H in a dense and compacted matrix. This can be attributed to the nucleation site effect of the NS particles. Figure 8d shows the dense structural matrix of the 0.0375% NCel mix, filled with the C–S–H component. This can be attributed to the nucleation effect of the NCel, as well as the role of NCel fibers in impeding the progress of cracks.

### The hybrid effect of the utilized nanomaterials on HESC

#### Compressive strength

**Fig. 9:**
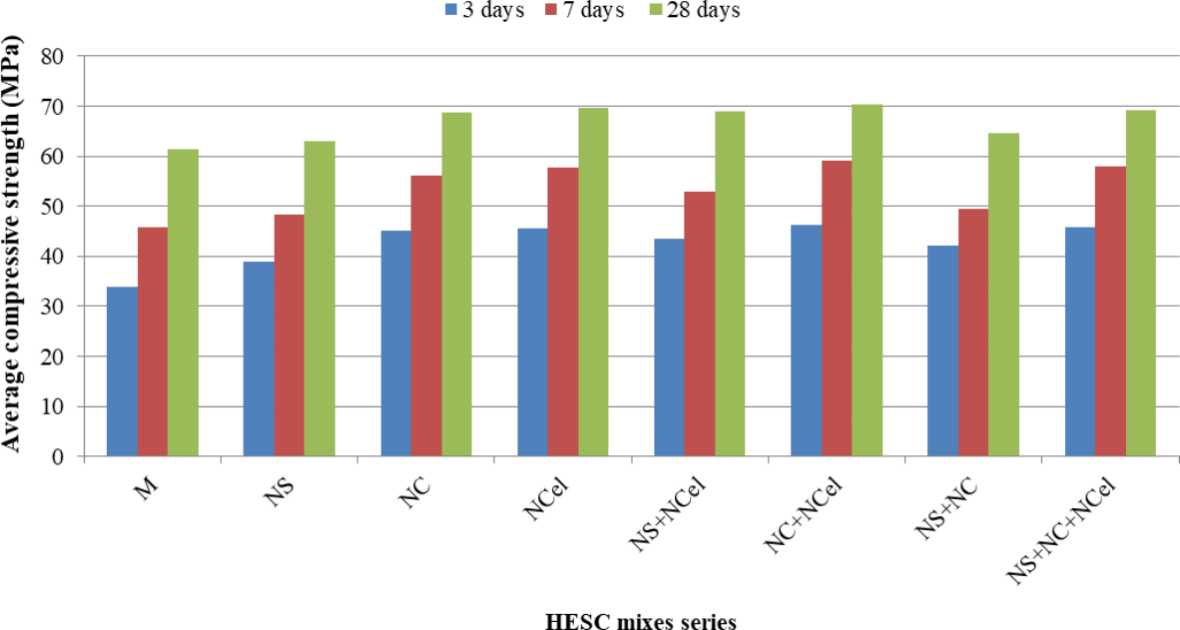
The compressive strength of HESC mixes.

Figure 9 presents the compressive strength results of sixteen HESC mixes at different ages of 3, 7, and 28 days. The compressive strength of all HESC mixes containing nanomaterials (NC, NS, and NCel), either as separate or hybrid components, is higher than the mix that did not contain any nanomaterials at all studied ages of 3, 7, and 28 days. The enhancement in the compressive strength through the use of nanomaterials can be attributed to one or more of the following effects on the matrix structure: (a) Filling effect of NC, NS, and NCel particles due to their nano-scale size, resulting in a uniform, highly compacted, and dense structured matrix^33–37,41–45,48^. (b) Pozzolanic reactivity of NS and NC, which react with the excess calcium hydroxide (CH) from cement hydration and produce additional Calcium-Silicate-Hydrate (C–S–H) gel^33–43^. (c) Nucleation effect; nano particles of NS, NC, and NCel acting as nucleation sites for cement hydration phases, resulting in the reinforcement of the cement hydration products^31,33,35,38,40–42,46–48^. (d) Needle-like action of NC and NCel particles, which are elongated and thin, leading to the reinforcement of the cement component^22,26,31,33,40,46–48^. When comparing the effect of adding each type of nano material separately, the matrix containing NCel had the higher enhancement than using either NS or NC individually. This can be attributed to the state of NCel as a viscous gel, which has higher dispersion ability and a larger surface area than NC or NS in powder form. The nanomaterials in powder form tend to agglomerate in large substitution percentages due to van der Waals forces, resulting in a failure to fill the small pores within the matrix^22,25,39,73^. The mixes containing the three types of nanomaterials had the highest compressive strength at 3, 7, and 28 days, with strength gains of approximately 35.7%, 26%, and 12.75%, respectively, compared to the mix without nanomaterials.

#### Statistical analysis

##### Model prediction

Table 4 shows the compressive strength results of 16 mixes of HESC after 3, 7 and 28 days of curing according to the experimental design sorted in Table 1. The analysis of variance of the full interaction models for the compressive strength results at 3, 7 and 28 days in the 2^3^ factorial designs were shown in Tables 5, 6 and 7. The summaries for three statistical models are shown in Table 8.

**Table 4:** The compressive strength results for 3, 7and 28 days corresponding to the experimental design.

Run	Nanomaterials (wt%)			Compressive strength (Fc) MPa		
	NC	NS	Ncel	3 days	7 days	28 days
M1	0.0	4.5	0.0000	39.0	48.5	63.0
M2	4.5	4.5	0.0375	45.6	58.0	69.3
M3	0.0	0.0	0.0000	33.6	46.0	61.4
M4	0.0	4.5	0.0375	44.0	53.0	69.1
M5	4.5	0.0	0.0375	46.0	59.2	70.2
M6	4.5	0.0	0.0000	45.2	55.6	68.9
M7	0.0	0.0	0.0375	45.3	57.7	69.4
M8	4.5	0.0	0.0375	46.3	59.0	70.4
M9	4.5	0.0	0.0000	45.0	56.5	68.6
M10	4.5	4.5	0.0000	42.2	49.7	64.7
M11	4.5	4.5	0.0375	45.1	58.0	69.0
M12	0.0	4.5	0.0375	43.0	53.0	69.0
M13	0.0	0.0	0.0000	34.0	45.6	61.2
M14	0.0	0.0	0.0375	45.9	57.5	69.7
M15	0.0	4.5	0.0000	39.0	48.2	62.9
M16	4.5	4.5	0.0000	42.0	49.4	64.3

**Table 5:** Factorial regression of 3 days compressive strength.

Source	DF	Adjusted Sum of squares	Adjusted Mean of squares	F-value	*P*-value
Model	7	253.740	36.249	298.96	0.000
NC	1	70.560	70.560	581.94	0.000
NS	1	0.123	0.123	1.01	0.344
NCel	1	106.090	106.090	874.97	0.000
NC*NS	1	11.902	11.902	98.16	0.000
NC*NCel	1	36.000	36.000	296.91	0.000
NS*NCel	1	6.502	6.502	53.63	0.000
NC *NS* NCel	1	22.563	22.563	186.08	0.000
Error	8	0.970	0.121	–	–
Total	15	254.710	–	–	–

**Table 6:** Factorial regression for 7 days compressive strength.

Source	DF	Adjusted Sum of squares	Adjusted mean of squares	F-value	*P*-value
Model	7	353.099	50.443	656.17	0.000
NC	1	80.551	80.551	1047.81	0.000
NS	1	23.281	23.281	302.84	0.000
NCel	1	195.301	195.301	2540.50	0.000
NC*NS	1	7.701	7.701	100.17	0.000
NC*NCel	1	6.126	6.126	79.68	0.000
NS*NCel	1	0.766	0.766	9.96	0.013
NC *NS* NCel	1	39.376	39.376	512.20	0.000
Error	8	0.615	0.077	–	–
Total	15	353.714	–	–	–

**Table 7:** Factorial regression for 28 days compressive strength.

Source	DF	Adjusted Sum of squares	Adjusted Mean of squares	F-value	*P*-value
Model	7	168.794	24.113	727.95	0.000
NC	1	24.256	24.256	732.25	0.000
NS	1	4.516	4.516	136.32	0.000
NCel	1	105.576	105.576	3187.19	0.000
NC*NS	1	10.726	10.726	323.79	0.000
NC*NCel	1	16.606	16.606	501.30	0.000
NS*NCel	1	0.226	0.226	6.81	0.031
NC *NS* NCel	1	6.891	6.891	208.02	0.000
Error	8	0.265	0.033	–	–
Total	15	169.059	–	–	–

**Table 8:** The summaries for three statistical models.

Model	Standard deviation	R-Squared	Adjusted R-squared	Predicated R-squared#
F_C_ @3 days	0.348	0.9962	0.9929	0.9848
F_C_ @7 days	0.277	0.9983	0.9967	0.9930
F_C_ @28 days	0.182	0.9984	0.9971	0.9937

From the full factorial model, the compressive strength values of for 3, 7, 28 days respectively can be predicted from the following mathematical Eqs. (2) to (4);

2 $$\begin{aligned} {\text{Fc}}_{{@{\text{3 days}}}} & = {33}.{8} + {2}.{\text{511 NC}} + {1}.{\text{1556 NS}} + {314}.{\text{67 NCel }}{-} \, 0.{4}0{\text{49 NC}}*{\text{NS }} \\ & \quad {-}{ 36}.{7}0{\text{ NS}}*{\text{NCel}}. - {43}.{\text{26 NS}}*{\text{NCel}}\, + \,{12}.{\text{51 NC}}*{\text{NS}}*{\text{NCel}} \\ \end{aligned}$$

3 $$\begin{aligned} {\text{Fc}}_{{@{\text{7 days}}}} & = { 48}.{8} + {2}.{\text{2778 NC}} + 0.{\text{5667 NS}} + {314}.{\text{67 NCel }}{-} \, 0.{\text{4469 NC}}*{\text{NS }} \\ & \quad {-}{ 51}.{\text{85 NS}}*{\text{NCel}}. - {42}.{\text{37 NS}}*{\text{NCel}}\, + \,{16}.{\text{527 NC}}*{\text{NS}}*{\text{NCel}} \\ \end{aligned}$$

4 $$\begin{aligned} {\text{Fc}}_{{@{\text{ 28 days}}}} & = { 61}.{3} + {1}.{\text{65556 NC}} + 0.{\text{3667 NS}} + {22}0{\text{ NCel }}{-} \, 0.{\text{2914 NC}}*{\text{NS}} \\ & \quad {-}{ 39}.{7}0{\text{NS}}*{\text{NCel}}. - {12}.{\text{74 NS}}*{\text{NCel}}\, + \,{6}.{\text{914 NC}}*{\text{NS}}*{\text{NCel}} \\ \end{aligned}$$

From the above three Eqs. (2) to (4), nano cellulose (NCel) had the greatest contribution to the strength of HESC, and its admixture greatly affects the compressive strength for 3, 7 and 28 days. This may be because the NCel used is a viscous aqueous gel and due to mixing in water, it has higher dispersion ability than the NS used or the NC used in the powder state.

##### Assessing the feasibility of a 2^3^ factorial design

The applicability of the predicted mathematical models was evaluated by using ANOVA analysis to determine the compressive strength value corresponding to the content of the utilized nanomaterials. As shown in Table 6, when comparing the R-squared values of each of the three models with their respective adjusted R-squared values, it was found that the values are very close, which proves that these models are appropriate. The predicted R-squared values are also very close to both the R-squared and adjusted R-squared values, indicating the feasibility of the predicted mathematical models to calculate the compressive strength of HESC at 3, 7, and 28 days.

**Fig. 10:**
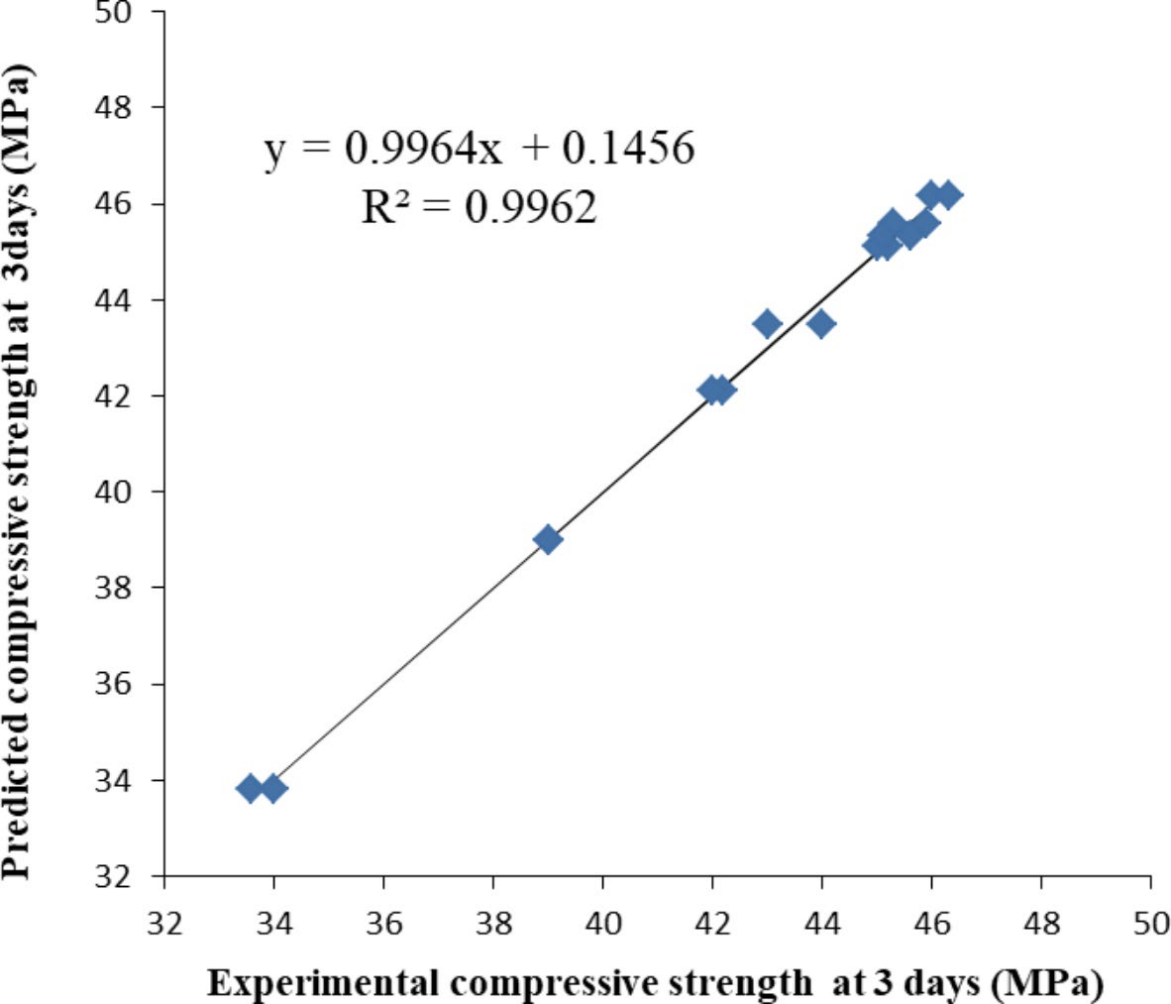
Relation between the experimental compressive strength results and the predicted compressive strength from mathematical equation at 3 days (the straight line is the predicted strength).

**Fig. 11:**
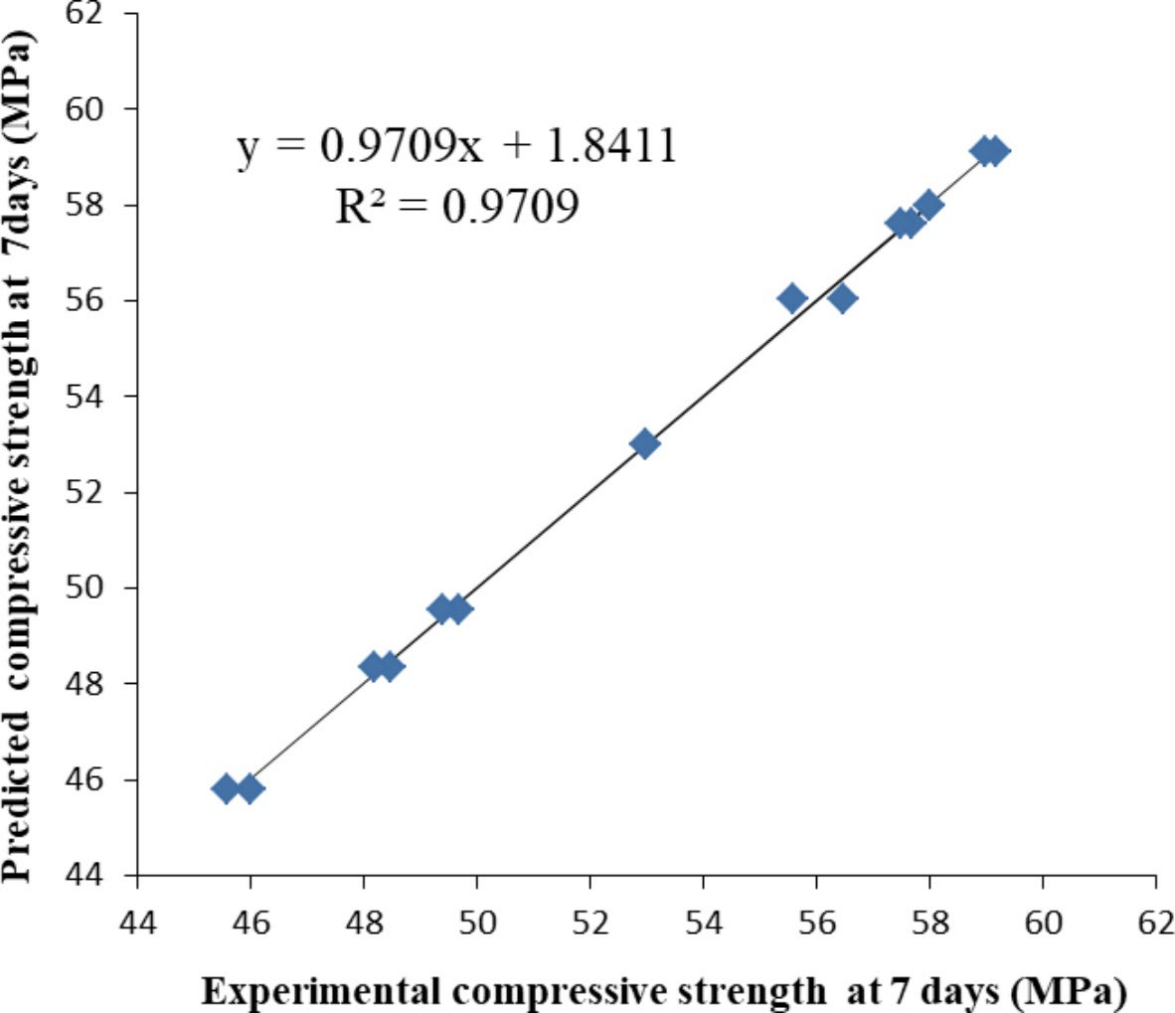
Actual measured compressive strength and predicted compressive strength at 7 days (the straight line is the predicted strength).

**Fig. 12:**
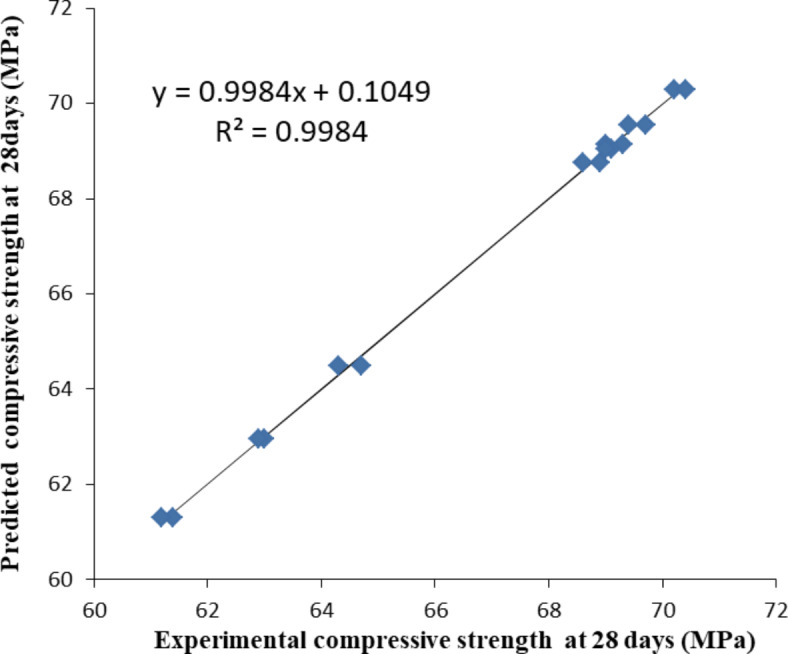
Actual measured compressive strength and predicted compressive strength at 28 days (the straight line is the predicted strength).

Figures 3, 4 and 5 illustrate the relationship between the experimental compressive strength results and the predicted compressive strength from the mathematical equations at 3, 7, and 28 days, respectively. The equations between the experimental compressive strength (X) and the predicted compressive strength (Y) are presented in Figs. 10, 11 and 12, with R^2^ = 0.9962, 0.9709, and 0.9984, respectively. The compressive strength results are closely surrounded on both sides of the straight line representing the predicted compressive strength values, indicating that the mathematical equations for the predicted compressive strengths are valid and closely match the compressive strengths obtained from the experiments.

**Fig. 13:**
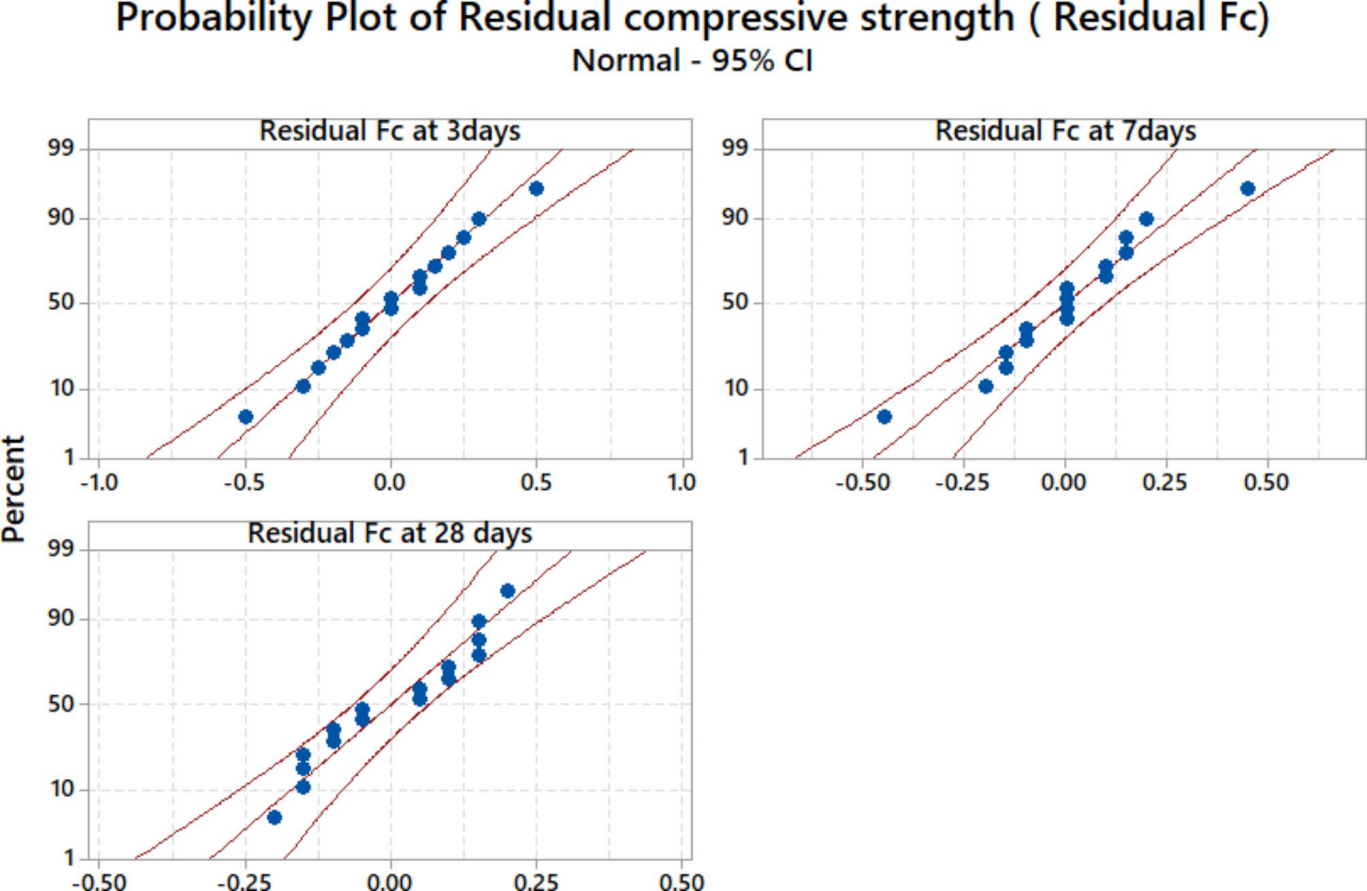
The normal distribution probability plot of the residual F_C_ at 3, 7, and 28 days.

Figures 13 shows the normal distribution probability plot of the residual compressive strength (Residual F_C_ = the difference between the experimental and predicted compressive strength) for different ages at the 95% confidence and 5% significance level interval. It was found the residual compressive strength values in the three models are normally distributed, which indicates that the mathematical equations to estimating the compressive strength are applicable and feasible.

##### Results of the model

**Fig. 14:**
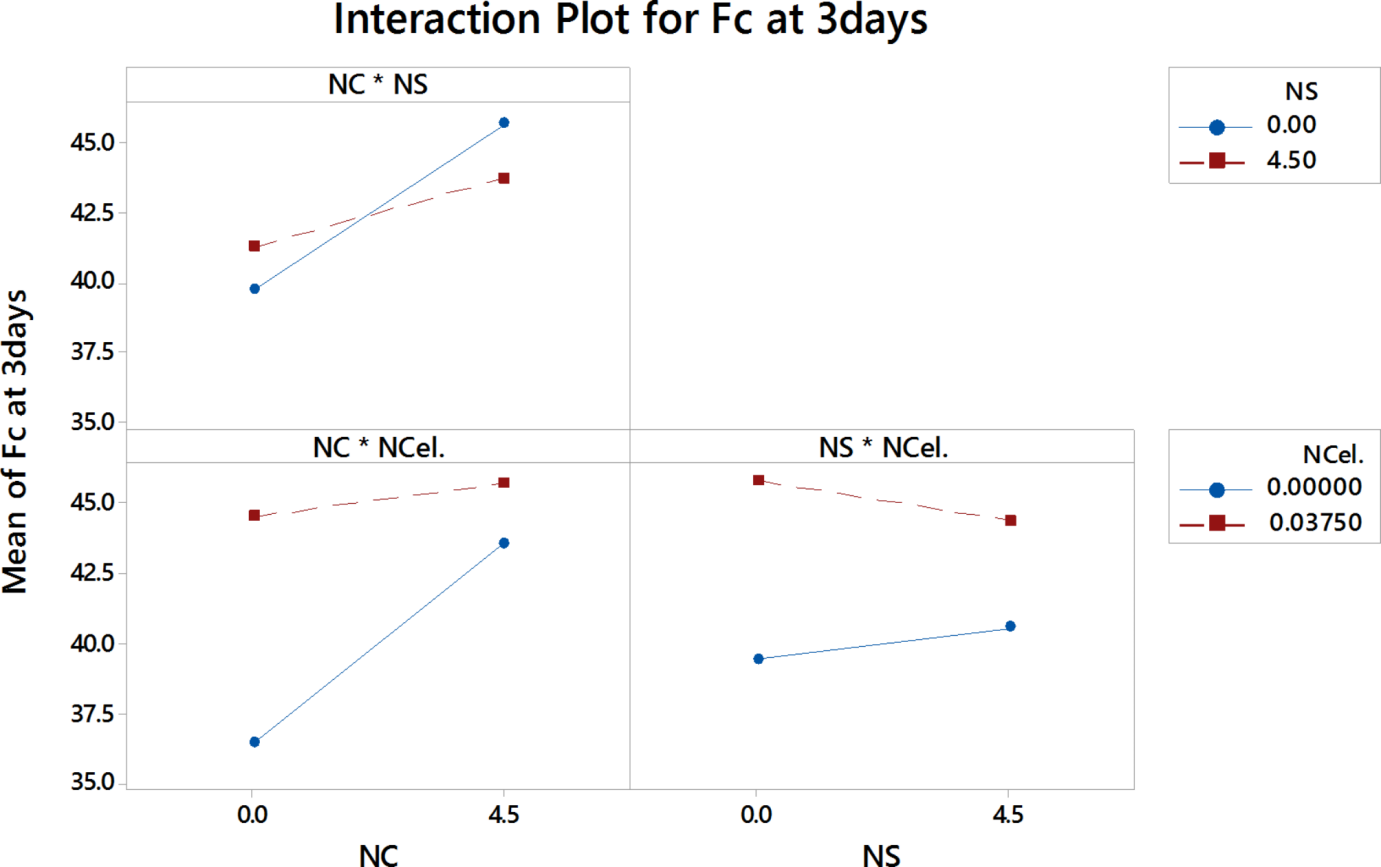
the interaction effect of three nanomaterials percentages on the F_C_ at 3 days.

**Fig. 15:**
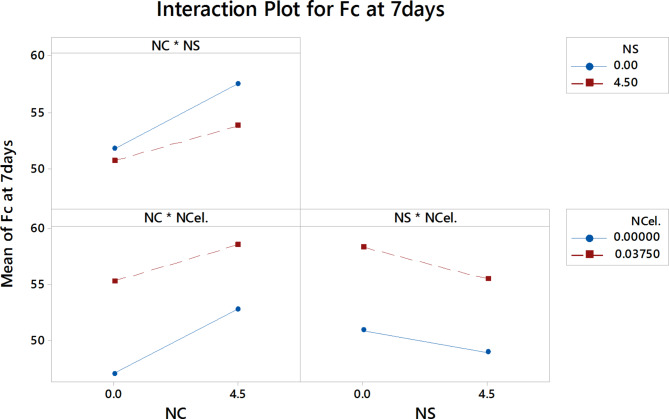
the interaction effect of three nanomaterials percentages on the F_C_ at 7 days.

**Fig. 16:**
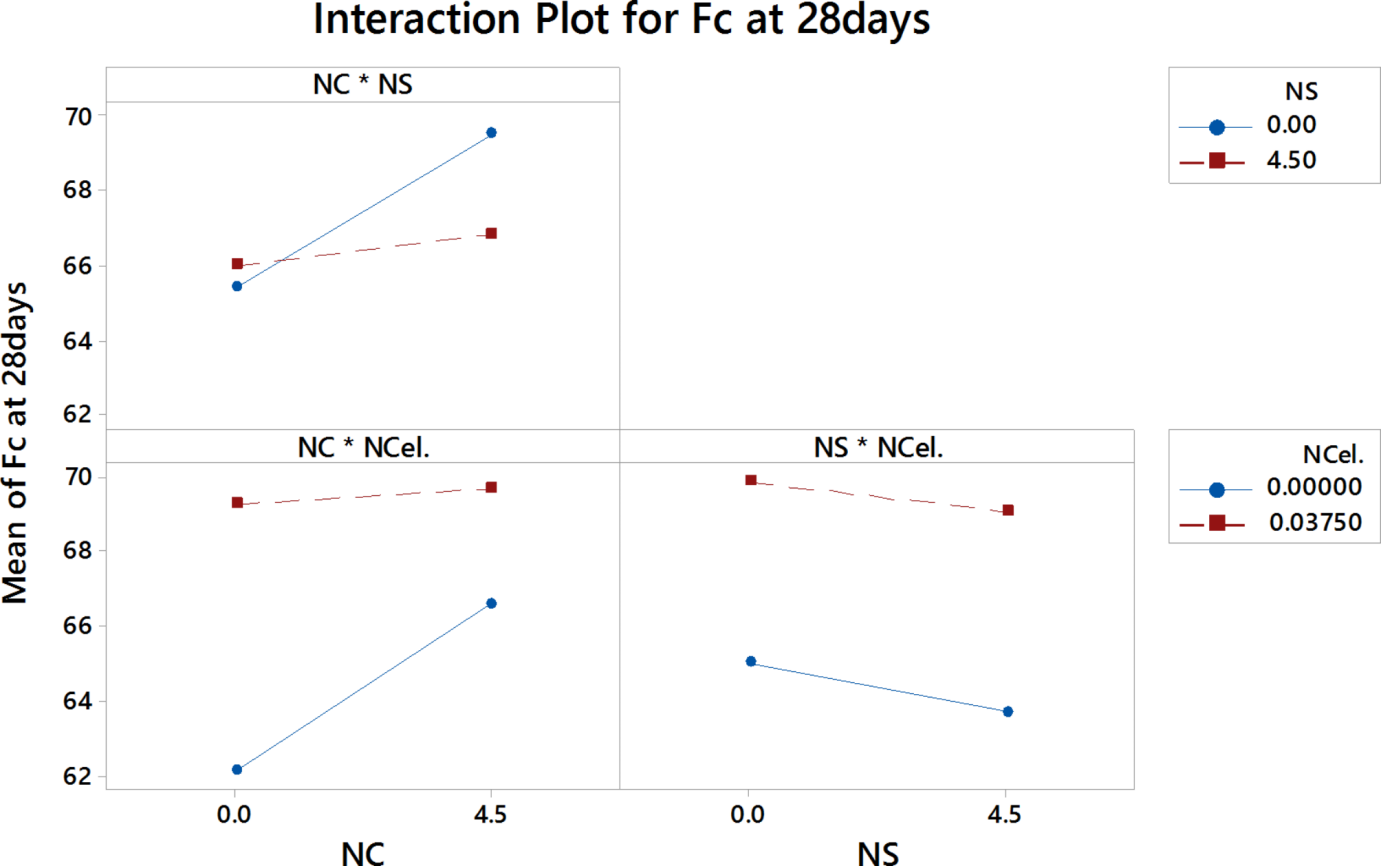
the interaction effect of three nanomaterials on the F_C_ at 28 days.

The interaction plot shows the effect of one variable on another variable and the effect of this on the studied response. Figures 14, 15 and 16 present the interaction effects for the compressive strength of HESC at 3, 7, and 28 days, respectively.

Figure 14 shows the interaction plots for each pair of nanomaterials as variables and the compressive strength at 3 days. According to the interaction plot of NC and NS, increasing NC percentages up to 4.5% separately led to enhancing the compressive strength of HESC at 3 days, which can be attributed to the high surface area of NC resulting in accelerated hydration at early ages, and its nucleation and pozzolanic effects^40–43^. However, at increasing the percentage of NS to 4.5%, the compressive strength at 3 days was enhanced at small percentages of NC and then the ability to enhance the compressive strength at 3 days was decreased with increasing the content of NC. This behavior is attributed to the tendency of nanoparticles to agglomerate at high percentages, resulting in not contributing significantly to enhancing the compressive strength, in agreement with^25,39,73^.

From the interaction plot of NC with NCel, the slopes of the two lines of NCel percentages are different, indicating a significant interaction between these NC and NCel percentages. It can be shown that increasing the percentage of either NC or NCel when used separately led to enhance the compressive strength at 3 days, while the 0.0375% NCel increases the compressive strength more than the 4.5% NC percentages. This can be attributed to the effect of the viscous gel state of NCel, which has a higher dispersion ability than NC, resulting in filling the nano-sized pores, in addition to the effect of NCel in accelerating cement hydration due to its high surface area, which acts as a nucleation site for CSH, resulting in a dense and uniform matrix and enhancing the compressive strength at 3 days^31,46–48^. The highest compressive strength can be obtained with the combination of high percentages of NC and NCel.

According to the interaction plot of NS with NCel, it can be shown that the line with 0% NCel trends slightly upward with increasing the NS percentages, while the line with 0.0375% NCel trends downwards with increasing the NS percentages. This can be attributed to the fact that increasing NS percentages up to 4.5% separately enhances the compressive strength, while with 0.0375% NCel, the increasing of NS particles led to decreasing the compressive strength. This can be attributed to the large agglomeration of NS particles that impeded the NCel particles from acting as a nucleation for cement hydration and filling the nano pores in the concrete matrix^72^.

Figure 15 shows the interaction plots for nanomaterials as variables and the compressive strength at 7 days. As shown in the interaction plot of NC and NS, increasing NC percentages up to 4.5% separately enhanced the compressive strength at 7 days significantly compared to the slight enhancement by increasing the NS percentages. This can be attributed to the poor dispersion of the nanoparticles in the powder state at high content^22,25,39,73^. The highest compressive strength at 7 days was achieved with 4.5% NC and 4.5% NS. According to the interaction plot of NC with NCel, it can be shown that the same trend appeared in the compressive strength at 3 days.

According to the interaction between NS and NCel, the two lines of the NCel percentages are parallel to each other, indicating that the interaction effects of both variables on the compressive strength at 7 days are not so significant. The presence of 0.0375% NCel with NS percentages enhanced the compressive strength compared to the presence of NS only, but the compressive strength reduced with increasing the NS percentages. This can be attributed to the large agglomerate of NS particles leading to a weaker matrix structure, while the presence of NCel particles that fill the small pores led to a denser matrix structure, resulting in increased compressive strength compared to the presence of NS only, in agreement with K.S. Kamasamudram^72^.

Figure 16 shows the interaction plots for nanomaterials as variables on the compressive strength at 28 days. The interaction effects between NS and NC and the interaction effects between NC and NCel showed the same trend as appeared in the 3-day interaction plots, while the interactions plots between the NS and NCel showed the same trend as the 7-day interactions.

**Fig. 17:**
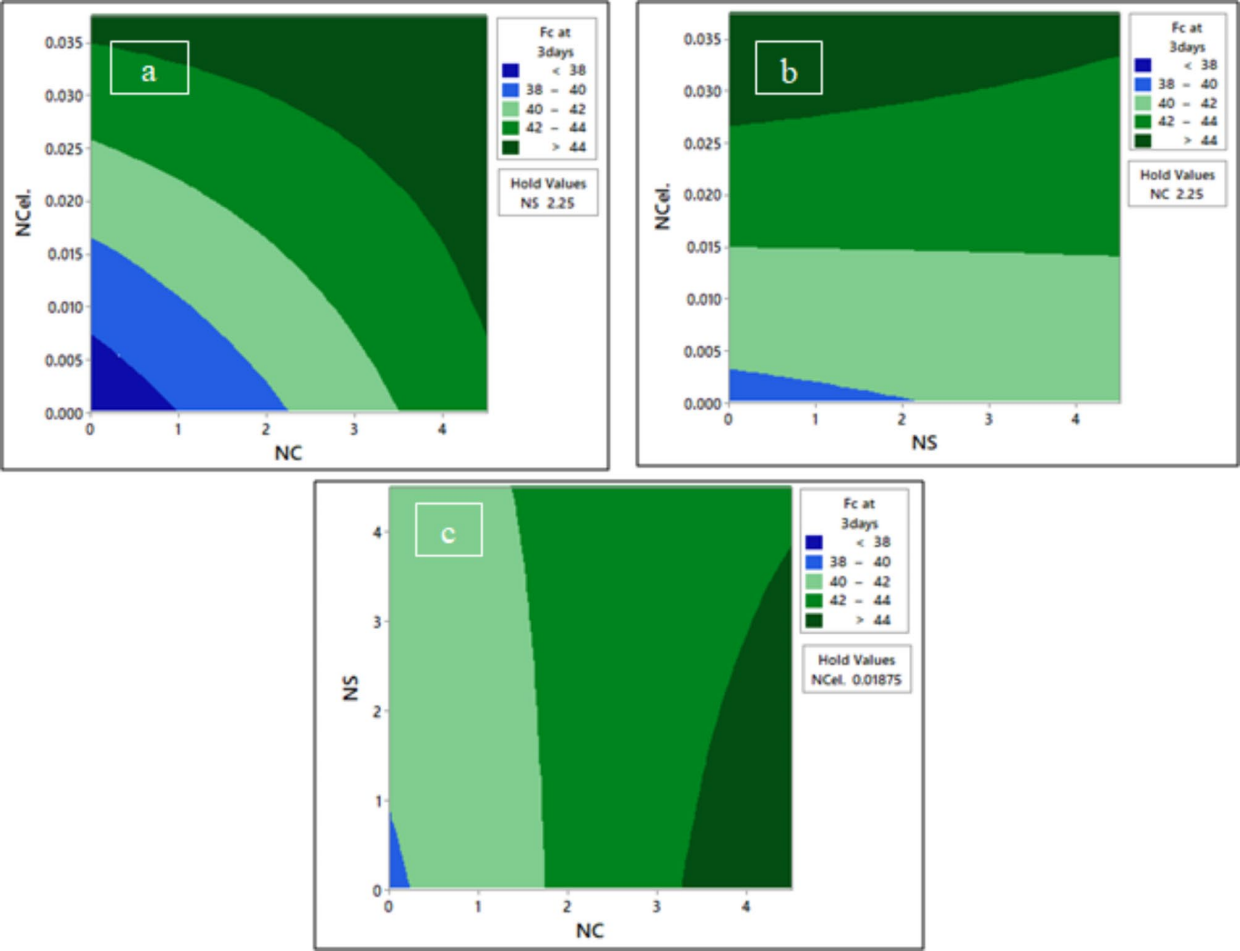
The contour plot of compressive strength F_C_ at 3 days; (**a**) F_C_ at 3 days vs NCel & NC, (**b**) F_C_ at 3 days vs NCel & NS, and (**c**) F_C_ at 3 days vs NS & NC.

**Fig. 18:**
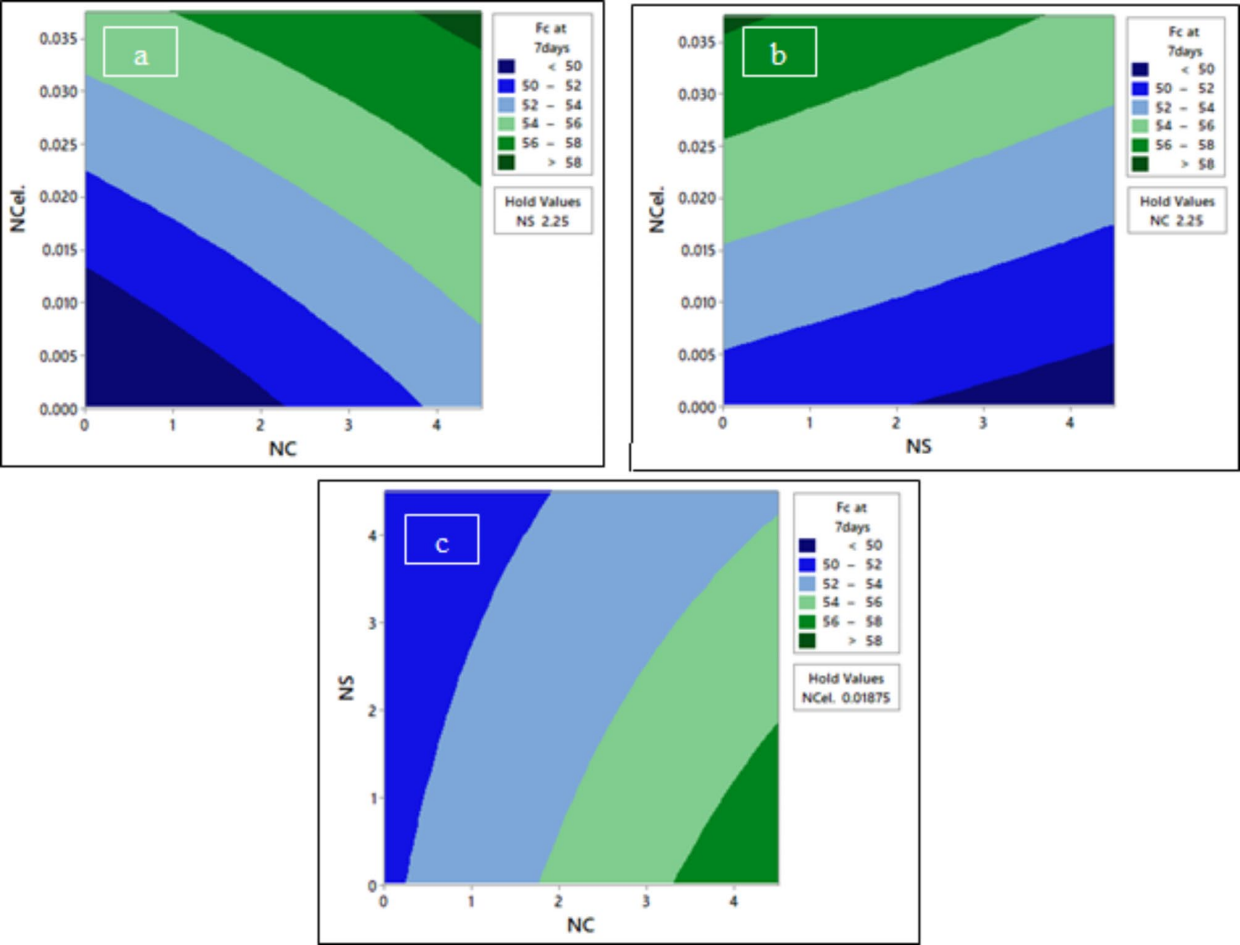
The contour plot of compressive strength F_C_ at 7 days; (**a**) F_C_ at 7 days vs NCel & NC, (**b**) F_C_ at 7 days vs NCel & NS, and (**c**) F_C_ at 7 days vs NS & NC.

**Fig. 19:**
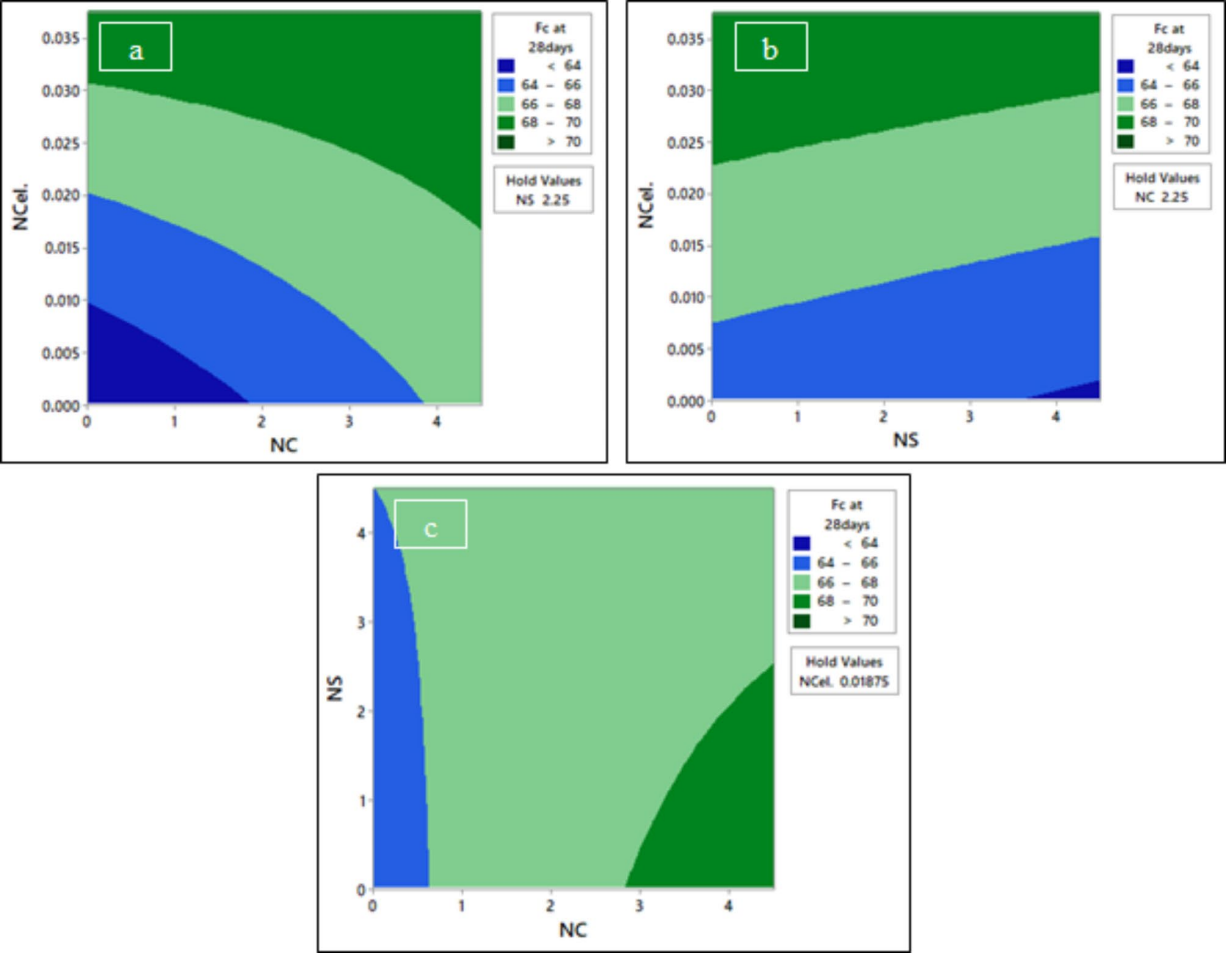
The contour plot of compressive strength F_C_ at 28 days; (**a**) F_C_ at 28 days vs NCel and NC, (**b**) F_C_ at 28 days vs NCel and NS, and (**c**) F_C_ at 28 days vs NS and NC.

Figures 17, 18 and 19 present the contour plots of the compressive strength of high-early-strength concrete (HESC) at 3, 7, and 28 days. The contour plots for the factorial model provide a two-dimensional view with two specified variables plotted on the horizontal and vertical axes, while the third variable is set at its average value to optimize the response.

From Fig. 17a, the compressive strength at 3 days can reach more than 44 MPa by using an NCel percentage greater than 0.035 without NC. To optimize the compressive strength to more than 44 MPa using NC and NCel together, it was found that increasing the content of NC led to decreasing the required NCel to 0.0055% at 4.5% NC. The compressive strength at 3 days can reach more than 44 MPa by using NCel at a higher percentage than 0.0252% without adding NS. When adding NS, the required percentage of NCel increases to 0.033%, as shown in Fig. 17b. At a fixed percentage of NCel at 0.01875%, as shown in Fig. 17c, the compressive strength at 3 days can be optimized by using a nano-silica percentage below 3.5% and an NC percentage greater than 3.3%.

From Fig. 18a, it can be seen that at a fixed percentage of 2.25% NS, the effect of using the NC percentage with NCel percentage on compressive strength at 7 days is directly proportional. In Fig. 18b, increasing the content of NCel and NC together led to a decrease in the compressive strength at 7 days. To optimize the compressive strength to reach more than 58 MPa, using an NCel percentage greater than 0.0355 and an NS percentage less than 0.5% is recommended. From Fig. 18c, it was observed that to reach the maximum compressive strength at 7 days with a fixed NCel content of 0.01875%, the nano-silica percentage should be below 1.8% and the NC percentage should be more than 3.4%.

Figure 19a shows that the compressive strength at 28 days followed the same trend as at 3 days. It was found that increasing the content of NC led to decreasing the required NCel to 0.018% at 4.5% NC when improving the compressive strength to range from 68 to 70 MPa. As shown in Fig. 19b, to maintain the compressive strength at 28 days to be more than 68 MPa, increasing the NS content up to 4.5% results in increasing the required NCel percentage from 0.023 to 0.03%. At a fixed NCel percentage of 0.01875%, as shown in Fig. 19c, the compressive strength at 28 days can be optimized to range from 68 to 70 MPa by using a nano-silica percentage below 2.5% and an NC percentage more than 2.8%.

## Conclusion

This paper contributes to the expanding knowledge base on nanomaterial-enhanced cementitious composites, offering valuable insights for developing high-performance, sustainable concrete solutions. The study assessed the effects of three different types of nanomaterials—nano clay (NC), nano silica (NS), and nano cellulose (NCel)—on the compressive strength of high-early-strength concrete (HESC) through both experimental studies and a 2^3^ factorial design. According to the test results and statistical analysis, the following conclusions were detected:

- Incorporating nanomaterials into the HESC matrix led to a decrease in workability, with NCel demonstrating the least impact on this property across all studied replacement percentages.
- All HESC mixes containing nanomaterials exhibited higher compressive strength than the control mix (M mix) across all ages. The optimal percentages for compressive strength enhancement were 4.5% NC (33.43% increase at 3 days, 22.29% at 7 days, and 12.15% at 28 days), 4.5% NS (20.12%, 11.14%, and 4.89% respectively), and 0.0375% NCel (34.91%, 25.76%, and 13.46% respectively).
- The highest compressive strength was observed in the hybrid mix containing 4.5% NC and 0.0375% NCel, yielding strength enhancements of 35.7%, 26%, and 12.75% compared to the control mix.
- Statistical analysis indicated that nano cellulose had the most significant contribution to enhancing compressive strength, followed by nano clay.
- The mathematical models derived from the statistical analyses provide a reliable means of predicting the compressive strength of HESC at 3, 7, and 28 days based on nanomaterial content.
- Contour plots illustrated the optimization of compressive strength across different nanomaterial contents at each age.

In summary, the findings underscore the potential of waste-derived nanomaterials to enhance the performance of HESC, paving the way for innovative waste utilization strategies in construction. The study emphasizes the importance of reducing curing times, improving structural durability, and minimizing the environmental impact associated with concrete production.

## Data Availability

All data generated or analyzed during this study are included in this published article.

## Abbreviations

Nc: Nano clay
Ns: Nano silica
Ncel: Nano cellulose
HESC: High-early-strength concrete
UFMA: Ultra-fine mineral admixture
